# A Diagnostic Dilemma: Atypical Systemic Pyoderma Gangrenosum

**DOI:** 10.7759/cureus.38763

**Published:** 2023-05-09

**Authors:** My Linh D Vu, Fiona E Lin, Cody R Ashcroft, Seth J Van Der Veer, Jacob R Hall

**Affiliations:** 1 Internal Medicine, Brooke Army Medical Center, San Antonio, USA; 2 Rheumatology, Brooke Army Medical Center, San Antonio, USA; 3 Dermatology, Brooke Army Medical Center, San Antonio, USA

**Keywords:** severe sepsis, multifocal pulmonary consolidation, paracelsus, neutrophilic dermatosis, idiopathic pyoderma gangrenosum

## Abstract

Pyoderma gangrenosum (PG) is a rare neutrophilic dermatosis that classically presents with chronic ulcerations with raised, violaceous, and undermined borders commonly found on the lower extremities. Less common presentations include tender nodules, pustules, or bullae that may occur on other sites of the body. In rarer circumstances, PG can lead to a systemic inflammatory response syndrome with extensive pulmonary infiltrates but ultimately cause and etiology of the disease are still uncertain. Unfortunately, there is no laboratory test or histopathologic finding that is specific to PG, which makes the diagnosis even more elusive.

## Introduction

Though first described in 1930, the etiology and pathogenesis of pyoderma gangrenosum (PG) remain poorly understood. It was initially named pyoderma due to the belief that it was related to hemolytic *Streptococcus* and *Staphylococcus* infections [1]. PG is rare and its incidence is estimated to be 3-10 per million people per year worldwide [2]. The exact mechanism of disease continues to be unclear, but hypotheses of disease etiology include neutrophil and cytokine dysregulation, auto-inflammatory, and even potentially genetic [3]. It is strongly correlated with systemic inflammatory diseases with approximately 50% of PG cases seen in patients with inflammatory bowel disease; up to 25% of cases of patients have rheumatoid arthritis, and about 7% of patients have a hematologic malignancy [3,4]. PG is typically treated with immunomodulatory drugs suggesting an immune-mediated mechanism [5,6]. The diagnosis of PG is difficult to establish and is largely a diagnosis of exclusion since there are no specific laboratory tests and the histopathology is indicative but not diagnostic [7].

Currently, there are five clinical variant types of PG: classical (ulcerative), bullous, pustular, vegetative, and peristomal [3]. Though it most commonly presents as a skin lesion, extracutaneous manifestations have been reported as well including lung, spleen, and eyes (i.e. corneal ulcerations and scleritis) [6]. Here, we discuss an atypical presentation of PG to further emphasize the elusiveness of the disease and its manifestations.

## Case presentation

**Figure 1:**
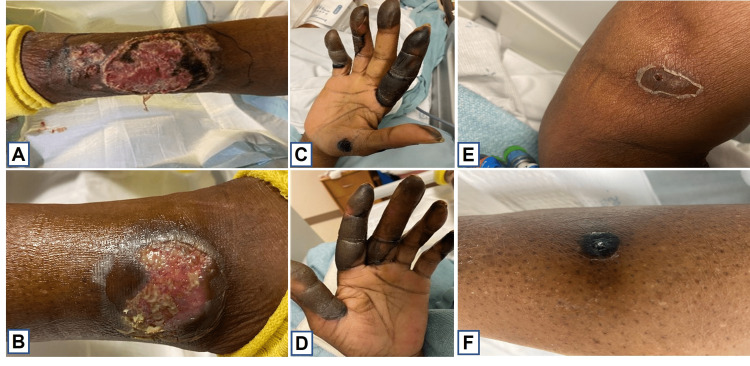
(A) Ulcerated lesion of left lateral lower extremity; (B) Ulcerated lesion of right lateral lower extremity; (C) Bullous, hyperpigmented lesions of distal right upper extremity and digits; (D) Bullous, hyperpigmented lesions; (E) Pustular lesion of right upper extremity; (F) Pustular lesion of lower extremity.

**Figure 2:**
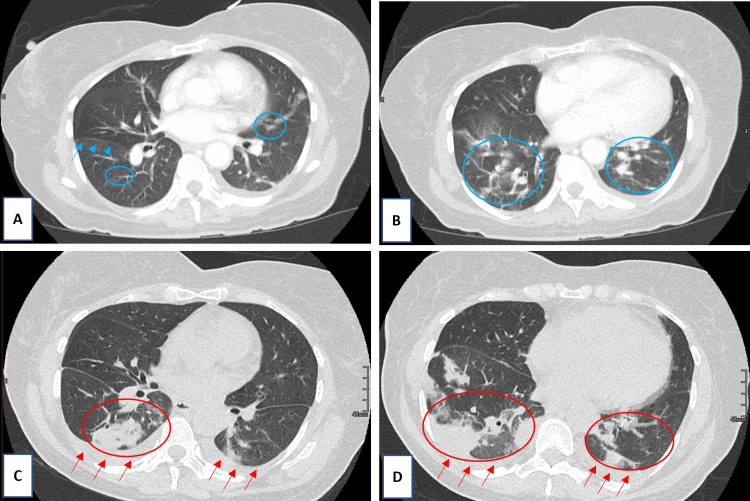
CT Chest serial axial images with A-B taken on initial evaluation (top row) and C-D taken on hospital day 4 with progression of multifocal lobar consolidation (bottom row). (A) Early right lower lobe perihilar ground-glass opacity (blue circles) and tracking along right middle lobe fissure (blue arrows); (B) Multifocal bilateral lower lobe consolidations (blue circles); (C) Formation of new right lower lobe consolidation (red circle) and progression of perihilar consolidation and bilateral pleural effusion (red arrows); (D) Interval worsening of bilateral lower lobe consolidation (red circles) and bilateral pleural effusion (red arrows).

A 65-year-old African American female with minimal past medical history presented to the Emergency Department for persistent fevers and progressively worsening painful bullae/pustules on her legs and fingers over three weeks concerning for sepsis (Figure 1). She was sent to the Emergency Department by her primary care provider out of concern for the failure of outpatient treatment of lower extremity cellulitis. Three weeks prior, she was prescribed cephalexin for her presumed lower extremity cellulitis, and when symptoms did not improve, she was started on clindamycin. However, antibiotics failed to improve the painful ulcerations on her legs. Two weeks prior to her admission she was also treated for presumably strep throat with intramuscular penicillin. Within the first 48 hours of admission, findings were significant for fevers to 101.3°F, tachycardia to 148 beats per minute, leukocytosis of 20.35, and CT of chest demonstrating multifocal pulmonary consolidations (Figure 2A, 2B). Punch biopsy of the lower left leg lesion showed non-specific sub-dermal neutrophilic bullous dermatosis with surrounding fibrinoid necrosis and no signs of vasculitis. Tissue cultures were negative for infection as well as periodic acid-Schiff for fungus (PAS-F) staining was negative for fungus. 

Overall, her initial presentation of multifocal pustular painful lesions was concerning for systemic infections such as disseminated gonorrhea or infective endocarditis. Transthoracic and transesophageal echocardiograms were performed to evaluate for infective endocarditis but were negative for valvular vegetations. Ultimately, no source of infection was established since all blood cultures and additional abdomen and pelvic imaging were also unremarkable. Additionally, an extended course of empiric broad-spectrum antibiotics including vancomycin, piperacillin/tazobactam, and doxycycline did not improve her clinical condition. A thorough workup for potential underlying autoimmune rheumatic disease was also performed and was similarly unremarkable. She was noted to have elevated rheumatoid factor; however, this can also be falsely elevated in the setting of an inflamed state.

**Figure 3:**
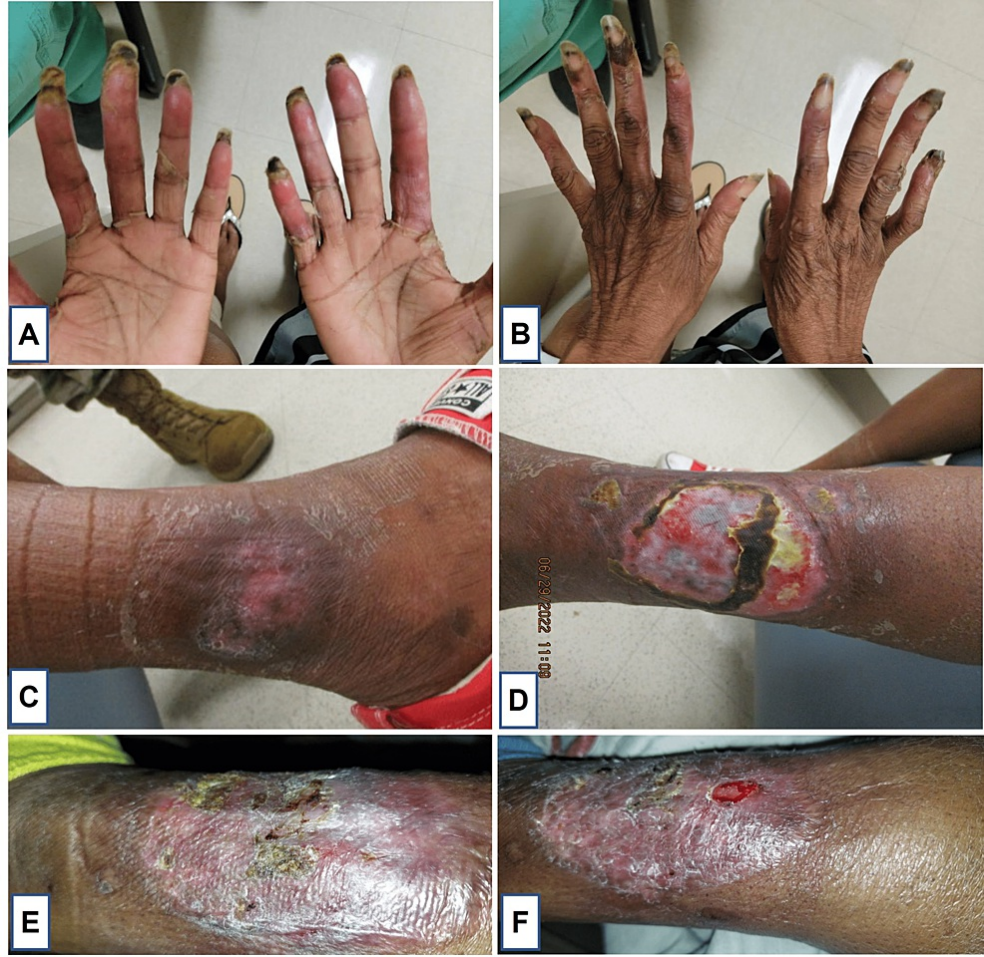
Improvement of bilateral hands and lower extremity lesions during steroid taper at 40 days (A-D) and remaining wound at six months following initiation steroids (E-F). (A) Bilateral palms; (B) Bilateral dorsa of hands; (C) Lateral lower right extremity; (D-F) Lateral left lower extremity.

On hospital day three, she was briefly transferred to the medical intensive care unit (MICU) for worsening hemodynamic status with persistent sinus tachycardia to 150s, febrile to 103.7°F, and mildly decreased mentation and responsiveness. In the MICU, she also received a bronchoscopy with cultures collected that ultimately were all negative for any infectious etiology before returning to the medicine floor after stabilization. On hospital day four, her pulmonary status deteriorated to the point of requiring supplemental oxygen prompting repeat chest CT showing progression of multifocal infiltrates (Figure 2A-2D). This was the turning point in her case when we considered the pulmonary involvement and systemic inflammatory response as systemic features of a neutrophilic dermatosis. When considering the appearance of the ulcerative lesions on her lower extremities, as well as the recent pathergy reactions to skin biopsy, atypical pyoderma gangrenosum was most likely. After a multidisciplinary discussion with Dermatology, Pathology, Hematology/Oncology, Rheumatology, and Infectious Disease, she was started on daily intravenous methylprednisolone 60 mg for the treatment of atypical PG with systemic features. Within 24 hours, she experienced a dramatic improvement and resolution of her tachycardia, tachypnea, and oxygen requirement. The bullous lesions on her hands and the ulcerated lesions on her legs improved daily, and she was subsequently discharged with a prolonged steroid taper and wound care. She continued to have an improvement in wounds after discharge with steroid therapy (Figure 3).

After discharge, she was followed up with Gastroenterology, which was notable for the resolution of her elevated liver-associated enzymes that were most likely caused by drug-induced liver injury given her long course of various antibiotics while hospitalized. Otherwise, her hepatitis panel, autoimmune hepatitis, and colonoscopy were all unremarkable and she had no gastrointestinal symptoms to suggest inflammatory bowel disease or colon cancer. Rheumatologic serologies have also been unremarkable with negative antinuclear antibody (ANA), anti-mitochondrial antibody, and anti-smooth muscle antibody as well as she lacks clinical symptoms for vasculitis given no upper respiratory, ocular, or renal involvement. Hematologic workup to date has been negative for any active forms of cancer. During her admission, she had elevated kappa and lambda free light chains at 54.3 mg/L and 55.9 mg/L, respectively. However, the kappa/lambda ratio was 0.97, therefore within normal limits and elevated free light chains is more likely a reflection of systemic inflammation as an acute phase reactant and less likely to suggest monoclonal gammopathy. No M-spike was noted on serum protein electrophoresis, ruling out multiple myeloma. Therefore, a bone marrow biopsy was performed after discharge but was unremarkable for hematologic malignancy. Though interestingly, molecular gene testing showed translocation of 11;14, which has been associated with favorable-risk multiple myeloma. However, her serum protein electrophoresis was negative for elevated M-spike and does not meet the criteria for multiple myeloma at this time. 

## Discussion

This case demonstrates the importance of maintaining a broad differential in diagnosing a condition as rare as systemic PG. In recent years, three diagnostic frameworks were compared in diagnosing PG and found the PARACELSUS score to be the most accurate with 89% accuracy while the Su and Delphi criteria both identified 74% of PG patients. By PARACELSUS, diagnosis of PG requires fulfillment of at least 10 points from three major criteria: rapidly progressing disease, assessment of other relevant differential diagnoses, and wound with reddish-violaceous border (3 points each), four minor criteria: alleviation with steroids, irregular ulcer shapes, extreme pain, and pathergy (2 points each), along with three additional criteria: suppurative inflammation, undermined wound borders, and associated systemic disease (1 point each) [8]. In our case, the patient fulfilled all criteria aside from systemic disease resulting in 19 out of 20 points, and, therefore, the disease was most consistent with PG over other neutrophilic dermatoses such as Sweet Syndrome.

## Conclusions

The patient’s condition was eventually diagnosed and treated, but it required multi-disciplinary collaboration to appropriately rule out other possible etiologies such as but not limited to disseminated gonococcal infection, infective endocarditis, hematologic and solid malignancy, and various vasculitis. This case also illustrates several atypical features that complicated and slowed diagnosis including lung involvement, systemic symptoms, and less common presentation with pustular and bullous lesions. Most interestingly, our case occurred in the absence of underlying rheumatic, enteropathic, hematologic, or oncologic disorders. Finally, given the current limitations with lack of diagnostic histologic and laboratory findings, it reflects the need for further research to refine diagnostic frameworks for PG for earlier management as it can be difficult to distinguish in presence of atypical features and lack of associated underlying disease such as our patient.
